# Arbuscular Mycorrhizal and *Trichoderma longibrachiatum* Enhance Soil Quality and Improve Microbial Community Structure in Albic Soil Under Straw Return

**DOI:** 10.3390/jof11100747

**Published:** 2025-10-18

**Authors:** Yu Zhang, Bo Zhang, Qiuju Wang, Jie Liu, Wenwen Xun, Yanling Lv, Fuqiang Song, Hongjiu Yu

**Affiliations:** 1 Engineering Research Center of Agricultural Microbiology Technology, Ministry of Education & Heilongjiang Provincial Key Laboratory of Ecological Restoration and Resource Utilization for Cold Region & Key Laboratory of Microbiology, College of Heilongjiang Province & School of Life Sciences, Heilongjiang University, Harbin 150080, China; zy06122022@163.com (Y.Z.); wbl970512@163.com (B.Z.); 2 Heilongjiang Academy of Black Soil Conservation & Utilization, Heilongjiang Academy of Agricultural Sciences, Harbin 150086, China; bqjwang@126.com (Q.W.); liujie1677@126.com (J.L.); 3 Agricultural Technology Extension Center of Fujin City, Jiamusi 154002, China; 18145116957@163.com (W.X.); elszyl@163.com (Y.L.)

**Keywords:** AMF, microbial inoculant, soil enzyme activity, microbial function prediction, soil quality index (SQI)

## Abstract

Albic soil is acidic and nutrient-deficient, resulting in low soil fertility that significantly limits the sustainable utilization of arable land. Microbial inoculants have emerged as promising biotechnological interventions to enhance soil quality by stimulating microbial activity and facilitating nutrient accumulation. This study focused on improving the characteristics of albic soil through the incorporation of straw residues combined with inoculation treatments involving arbuscular mycorrhizal fungi (AMF) and *Trichoderma*. Four experimental treatments were established: a Control (CK), single inoculation with AMF (AM), single inoculation with *Trichoderma* (TL), and a combined inoculation of both (AT). The investigation focused on assessing the synergistic effects of the AT treatment on albic soil chemical characteristics and its microbial community structure. The AT treatment significantly elevated rhizosphere soil pH, TN, and TP by 3.8%, 19.2%, and 10.9%, respectively, relative to the Control. The AT treatment markedly enhanced soil cellulase, β-glucosidase, and lignin peroxidase activities by 24.9%, 77.6%, and 36.7%, respectively, and increased the SQI by 78.9% compared to CK. Furthermore, the AT treatment led to a higher correlation abundance of *Sphingomonas* and aerobic chemoheterotrophs taxa. Structural equation modeling (SEM) uncovered that the interactions between AMF and *Trichoderma* modulated microbial community functions, augmented soil enzyme activities, promoted nutrient accumulation, and consequently improved albic soil quality. This study elucidates arbuscular mycorrhizal and *Trichoderma longibrachiatum* interactions with the soil environment, providing insights for improving albic soil health and microbial community structure.

## 1. Introduction

Soil degradation is one of the core challenges constraining the sustainable development of global agriculture. Albic soils are distributed across 32 countries worldwide, and in China, they are primarily concentrated in the northeastern region, representing one of the typical degraded soil types [1]. This soil type severely limits global agricultural productivity. As a representative barrier soil, the restrictive properties of albic soils are primarily evident in two key aspects. Firstly, the humus layer is insufficiently thick, resulting in limited nutrient retention capacity. Secondly, the characteristic albic horizon exhibits acidity, low fertility, and a compact structure [2]. The diminished soil organic matter content and reduced microbial activity in albic soils substantially hinder nutrient cycling processes, thereby contributing to the degradation of arable land quality [3]. Straw return technology has been extensively implemented in agricultural production, which can increase total soil porosity and the proportion of effective pores, improve soil structure stability, and promote a more balanced soil three-phase system [4]. Concurrently, the application of microbial agent soil amendments has emerged as a sustainable bio-amendment strategy and a focal point of the current studies aimed at environmentally friendly soil enhancement [5]. Microbial agents have been shown to modulate soil nutrient cycling pathways and optimize the composition of microbial communities, thereby improving soil properties [6]. Consequently, the integration of microbial agents with straw return presents considerable potential for ameliorating albic soil characteristics, enhancing soil quality, and boosting agricultural productivity and efficiency [7].

Arbuscular mycorrhizal fungi (AMF) establish symbiotic associations with most terrestrial vascular plants, playing a pivotal role in promoting soil fertility and enhancing enzymatic activity [8]. The extraradical mycelium of AMF secretes various organic compounds, which can positively modulate the composition of rhizosphere microdomains and shape microbial community structure [9]. Moreover, AMF facilitate nutrient cycling and uptake during straw incorporation, thereby improving soil quality and contributing to ecosystem stability [10]. Studies on straw decomposition have shown that *Trichoderma* species efficiently degrade cellulose, optimize rhizosphere conditions, and elevate nutrient levels in the rhizosphere soil [11]. *Trichoderma longibrachiatum* Rifai, a fungus commonly present in soil and decaying wood [12], produces cellulases, xylanases, and proteases that effectively break down lignocellulosic biomass. It also significantly enhances rhizosphere enzyme activities and accelerates soil biochemical processes [13]. The interactions between AMF and *Trichoderma* have been found to modify soil chemical status, enhance soil fertility, and increase nutrient availability to plants [14]. Therefore, investigating the combined regulatory effects of AMF, as a symbiotic fungus, and *T. longibrachiatum*, as a saprotrophic fungus, on soil microbial communities and soil properties under straw-returning conditions is of considerable significance.

AMF and *Trichoderma* can simultaneously colonize plant roots to protect healthy root growth and increase root survival [15,16], thereby remarkably enhancing tomato seedling growth and yield [17]. Co-inoculation of AMF with *Trichoderma* significantly upregulated the expression of disease defense-related pathway genes, such as “isoflavone biosynthesis”, and reduced the incidence of root rot by 51% and 57.5% in CICR-3 and CSH-3129 cotton varieties, respectively [18,19,20]. Furthermore, the synergistic association of AMF with *Trichoderma* has been shown to promote more efficient nutrient uptake and boost plant resilience to soil salinity stress [21,22]. The majority of current studies predominantly address the enhancement of plant disease resistance and the amelioration of saline soils through the fungal agent. However, there remains a notable gap in this study on the improvement of albic soil and the modulation of microbial communities by the joint inoculation of AMF and *Trichoderma*.

Therefore, this study aimed to investigate the influence of single applications of *Funneliformis mosseae* and *Trichoderma longibrachiatum*, as well as their combined application, on soil quality and microbial community structure in albic soil under straw-returning conditions. We proposed two hypotheses: (i) AMF combined with *Trichoderma* enhances soil enzyme activities and promotes the accumulation of soil nutrients; (ii) AMF and *Trichoderma* reshape soil microbial community structure, accelerate organic matter decomposition and nutrient transformation, and thereby significantly improve albic soil quality. This study provides a scientific basis and theoretical reference for the use of microbial agents to enhance albic soil quality.

## 2. Materials and Methods

### 2.1. Experimental Setup, Test Materials

The experiment was conducted in 2024 from May to September in the core experimental area of No. 852 Farm of Beidahuang Group Co. Ltd. (132°38′40.387″ E, 46°14′30.783″ N) in Shuangyashan, Heilongjiang Province, China. This area is the primary distribution region of typical albic soil and features a cold-temperate continental monsoon climate, with yearly precipitation averaging 548 mm and a mean annual temperature of 3.2 °C. The basic chemical properties of the albic soil were pH 5.94, organic carbon 30.08 g·kg^−1^, total phosphorus 0.56 g·kg^−1^, total potassium 14.62 g·kg^−1^, total nitrogen 1.28 g·kg^−1^, available phosphorus 15.36 mg·kg^−1^, available potassium 133.51 mg·kg^−1^, and alkali-hydrolyzable nitrogen 164.73 mg·kg^−1^. The soybean variety used in this experiment was Kennong 34, which is a staple cultivar in this region and provided by No. 852 Farm of Beidahuang Group, Heilongjiang Province. The experimental plots were located in a soybean–maize rotation area, with maize as the preceding crop. Maize stover was collected, chopped into 2–3 cm pieces, and manually plowed back into soil within the upper 20 cm. The initial nutrient content of maize stover was organic carbon 365 g·kg^−1^, total phosphorus 1.14 g·kg^−1^, total potassium 5.85 g·kg^−1^, and total nitrogen 12.41 g·kg^−1^.

In this study, AMF were represented by *Funneliformis mosseae* (isolate number: CGMCC No. 3012), cultured in pots using sorghum sp. as a host with a spore density of 32 spores g^−1^. *Trichoderma* was represented by *T. longibrachiatum* Rifai (accession number: PV998066), cultured in liquid KM medium (Kaefer medium) with a concentration of 2.25 × 10^8^ CFU mL^−1^ (CFU, colony-forming unit).

The experiment was designed as a randomized block long-term positional trial, with plots measuring 10 m in length, 10 m in width, and covering an area of 100 m^2^. Four experimental treatments were established: a Control group without the addition of *F. mosseae* and *T. longibrachiatum* (CK); with the addition of *F. mosseae* (AM); with the addition of *T. longibrachiatum* suspension (TL); and with the combined addition of *F. mosseae* and *T. longibrachiatum* (AT). Full soybean seeds were selected for reserve, and they were soaked in a 3% hypochlorite solution for 10 min [23]. Subsequently, the seeds were rinsed five times repeatedly with sterile water and soaked at 4 °C for 12 h. Finally, they were stored away from light for 48 h to break seed dormancy [24]. The plots were arranged in a completely randomized design, each with an area of 4 m^2^ and a straw return rate of 4 kg per plot (based on the average biomass of 10,000 kg·ha^−1^ commonly returned to fields in Northeast China). Each treatment was replicated four times, totaling 16 plots. Basal fertilizer was applied at levels of 102 kg N ha^−1^, 82.8 kg P_2_O_5_ ha^−1^, and 72 kg K_2_O ha^−1^, with an additional topdressing of 69 kg N ha^−1^ pure at the nodulation stage. The plots were manually furrowed and sown, and the PI and BP treatments were inoculated with *T. longibrachiatum* inoculum at a standard mycelial suspension dosage of 1 L per plot. It was necessary to first dilute 8 L of the mycelial suspensions containing conidia to 80 L with sterile water. To prevent the potential confounding effects of nutrients from the growth medium, equivalent volumes of sterilized fungal suspension were added to CK and BC treatments. The seeds were sprayed with distilled water and then gently stirred with the AMF inoculation until stuck to the surface of the soyabeans. The mixing ratio was inoculum–seed = 1:5 (*w*/*w*), and seeds were sown within 12 h to prevent loss of fungal viability. Soybean seeding rate was calculated as 6 kg per acre, and the AMF inoculum addition rate was 1.2 kg per acre. Soil samples were collected at soybean harvest in late September 2024. Rhizosphere soil closely adhering to the soybean roots was gently brushed off and retained. From each replicate, 30 g of soil was collected. The gently brushed rhizosphere soil was used for 16S and ITS rRNA gene sequencing and soil enzyme activity analysis. The firmly adhered soil shaken from the roots was air-dried for determining soil chemical properties, with 50 g per replicate being reserved.

### 2.2. Soil Chemical Properties Determination

Soil pH was determined by applying the pH meter at soil-to-water ratio of 1:2.5. Soil organic matter (SOM) was quantified by the high-temperature exothermic K_2_Cr_2_O_7_ volumetric method [25]. Total phosphorus (TP) was measured by alkali fusion-molybdenum antimony colorimetric method [26]. Total potassium (TK) was surveyed by NaHCO_3_ fusion followed by flame photometry [27]. Available phosphorus (AP) was extracted with NaHCO_3_ and determined by the molybdenum antimony colorimetric method [27]. Alkali-hydrolyzable nitrogen (AN) was determined by alkaline distillation [27]. Total nitrogen (TN) was measured using the digestion–distillation method with K_2_Cr_2_O_7_-H_2_SO_4_ as the digestion reagent [28]. Available potassium (AK) was determined by flame photometry using ammonium acetate (CH_3_COONH_4_) as the extractant [27]. Soil nitrate nitrogen (NO_3_^−^-N) was measured using a continuous flow analyzer based on the phenol disulfonic acid colorimetric method [29]. Soil ammonium nitrogen (NH_4_^+^-N) was determined using a continuous flow analyzer based on the indophenol blue colorimetric method [29].

### 2.3. Soil Enzyme Activities Determination

Soil enzyme activities were determined using commercially available assay kits from Beijing Solarbio Science and Technology Co., Ltd. (Beijing, China). The specific enzymes and corresponding kit models were as follows: soil β-glucosidase (S-β-GC) using kit BC0165, soil urease (S-UE) using kit BC0125, soil cellulase (S-CL) using kit BC0155, and soil lignin peroxidase (S-Lip) using kit BC1615. All enzyme activities were determined according to the instructions of manufacturers.

### 2.4. Soil Quality Index Calculation

To derive the soil quality index (SQI), the Minimum Data Set (MDS) was utilized. In this study, Principal Component Analysis (PCA) and correlation analysis were combined to select key soil indicators from the Total Data Set (TDS) for the MDS. Components with a characteristic value ≥ 1 were retained, while soil indicators at loadings ≥ 0.5 on the same principal component were classified together. After grouping, Norm values for each indicator were calculated (Equation (1)), and those indicators showing Norm values no more than 10% below the maximum in each group were contained in the MDS. Pearson’s correlation analysis was applied to assess relationships between data. If a significant correlation (*p* < 0.01) was detected between indicators, only the maximum Norm value indicator was selected for inclusion in the MDS [30].

Indicators with *p*-values > 0.5 were regarded as strongly correlated, and those with the uppermost exemplar values were retained in the MDS. When *p*-values < 0.5, whole corresponding indexes were included in the MDS [31]. The variables pH, TP, TN, TK, AP, AK, S^_^β^_^GC, S^_^UE, S^_^CL, and S^_^Lip were selected for analysis in this study, which were selected as vital parameters of soil quality assessment, and corresponding SQI values were calculated using Equation (2) [32].

(1) $$N_{\mathrm{ik}}=\sqrt{\sum_{i=1}^{k}\left(u_{ik}^{2}\cdot e_{k}\right)}$$

where *N_ik_* represents the Norm value of the *i*th variable in the first *k* primary factors with characteristic value ≥ 1; *u_ik_* denotes the Norm value of the *i*th indicator in the kth primary factor; and *e_k_* indicates the characteristic value associated with the kth principal factor.

(2) $$SQI=\sum_{i=1}^{n}W_{i}\times F\left(X_{i}\right)$$

where *SQI* denotes the soil quality evaluation index; *W_i_* denotes the proportion associated with each evaluation index; and *F*(*X_i_*) represents the membership value of each assessment criterion.

### 2.5. Sequencing of Rhizosphere Microorganisms

The FastPure kit designed for soil DNA extraction was used to purify DNA. The purified DNA was first examined using 2% agarose gel electrophoresis to verify its quality, followed by measurement of its enrichment and purity. Then, integrity was evaluated by NanoDrop 2000 microvolume spectrophotometer (Thermo Scientific, Waltham, MA, USA). The purified DNA was subsequently used as a template for PCR amplification. For the bacterial 16S rRNA V3–V4 region, primers 338F (5′^_^ACTCCTACGGGGGAGGCAG^_^3′) and 806R (5′^_^GGACTACHVGGGTWTCTAAT^_^3′) were used. For the fungal ITS1 region (V4–V5), primers ITS1F (5′^_^CTTGGTCATTTAGAGAGAGGAAGTAA^_^3′) and ITS2R (5′^_^GCTGGTCTTCATCGATGC^_^3′) were employed [33].

The PCR reaction mixture (20 μL) included 10 μL 2× Fast Pfu buffer, 0.8 μL forward primer (5 μM), 0.8 μL reverse primer (5 μM), 0.4 μL Fast Pfu polymerase, 10 ng of template DNA, and ddH_2_O to a final volume of 20 µL. PCR amplification cycling conditions were as follows: initial denaturation at 95 °C for 3 min, followed by 27 cycles of denaturing at 95 °C for 30 s, annealing at 55 °C for 30 s and extension at 72 °C for 45 s, single extension at 72 °C for 10 min, and end at 10 °C. The PCR product was extracted from 2% agarose gel and purified using the PCR Clean^_^Up Kit (YuHua, Shanghai, China) according to manufacturer’s instructions and quantified using Qubit 4.0 (Thermo Fisher Scientific, USA). The purified PCR products were used to construct sequencing libraries using NEXTFLEX Rapid DNA^_^Seq Kit (Bioo Scientific, Austin, TX, USA). The constructed libraries were quantified and standardized and then subjected to high^_^throughput sequencing of 16S rRNA and ITS rRNA using the Illumina NextSeq 2000 platform at Shanghai Meiji Biomedical Technology Co. (Shanghai, China).

### 2.6. Data Processing

Soil chemical indicators in this study were expressed as mean and standard deviation. Experimental data were analyzed using Microsoft Excel and SPSS version 29.0. One^_^way ANOVA, two^_^way ANOVA, and Duncan’s multiple range test were applied to assess statistical significance. All statistical tests were performed to determine the significance of differences between groups at the *p* < 0.05 level, and the data passed the normal distribution and variance chi-square tests. Microbial community α^_^diversity was evaluated through indicators such as Chao1 and Shannon indices, which were assessed using the Wilcoxon rank-sum test in Mothur (1.30.2). β diversity was evaluated using PCoA derived from the Bray^–^Curtis dissimilarity matrix, implemented in the “factoextra” package of R (3.3.1). Selected top 20 genera by relative abundance were visualized as bar plots using Majorbio (https://cloud.majorbio.com/page/project/overview.html, accessed on 15 January 2025). Microbial lineage differentiation and biomarker identification were conducted using Linear Discriminant Analysis Effect Size (LEfSe, http://galaxy.biobakery.org/, accessed on 15 January 2025), with a significance threshold set at LDA > 3.5. Functional annotation of fungal and bacterial communities in albic soil was performed using FUNGuild (http://www.funguild.org/, accessed on 17 January 2025) and FAPROTAX (1.2.1), respectively, focusing on the top 15 genera based on relative abundance. The relative abundance of functional groups was determined from the mean Z^_^score of their constituent taxa. Redundancy Analysis (RDA) was conducted to explore how fungal and bacterial genera correlate with various soil chemical parameters, using the “vegan” package in R (3.3.1). All soil indicators of the Mantel test and Structural Equation Modeling (SEM) were standardized on a scale of 0–1. The Mantel test, based on the Pearson correlation coefficient, was applied to assess associations between microbial community construction and rhizosphere soil enzyme activities and chemical properties using the ecodist package in R (3.3.1). SEM was conducted by AMOS 22.0 (IBM, USA). Complete data visualization and figure preparation were carried out using GraphPad Prism 10 and Adobe Illustrator 2022.

## 3. Results

### 3.1. Arbuscular Mycorrhizal and T. longibrachiatum on Chemical Characteristics of Albic Soil

The different treatments notably improved soil pH, with the AT treatment being the greatest with a significant increase of 3.8% (Table S1). Furthermore, different inoculation treatments significantly increased soil TN and TP content. Compared to the CK treatment, the AM, TL, and AT treatments increased TN by 7.5%, 21.2%, and 19.2%, and TP by 3.1%, 15.6% and 10.9%, respectively. Notably, the two^_^way ANOVA results showed a significant interaction effect for SOM, TN, and TP across treatments. In addition, the AM and AT treatments markedly elevated TP content by 4.0% and 2.8%, respectively, compared to CK treatment. NH_4_^+_^N content was increased by 10.8%, 2.2%, and 14.0% in AM, TL, and AT treatments, respectively, in comparison with CK, but no statistically significant differences were observed.

### 3.2. Arbuscular Mycorrhizal and T. longibrachiatum on Soil Enzyme Activities and SQI in Albic Soil

**Figure 1:**
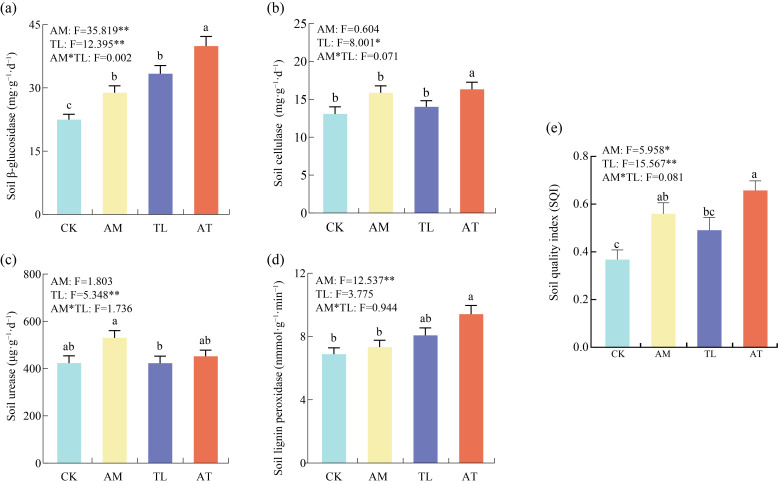
Effect of various treatments on enzyme activities and SQI. (**a**) Soil β-glucosidase activity; (**b**) soil cellulase activity; (**c**) soil urease activity; (**d**) soil lignin peroxidase activity; (**e**) soil quality index. CK: Control; AM: AMF application treatment; TL: *T. longibrachiatum* application treatment; AT: AMF and *T. longibrachiatum* application treatment. Different letters in the same row show statistically significant differences. * *p* < 0.05, ** *p* < 0.01.

S-β-GC activity significantly increased by 28.3% and 48.5% under the AM and TL treatments, respectively, in comparison with the CK. However, the variations among S-UE, S-CL, and S-Lip activities were not statistically significant (Figure 1a–d). Among all treatments, the AT treatment generated a notable enhancement in S-β-GC, S-CL, and S-Lip activities by 77.6%, 24.9%, and 36.7%, respectively, with the most pronounced impact observed on S-β-GC. Although all inoculation treatments elevated S-UE activity to some extent, none of the increases were statistically significant (Figure 1c). Two-way ANOVA revealed that inoculation with AMF had a significant effect on S-β-GC and S-Lip activities, while inoculation with *T. longibrachiatum* significantly affected S-β-GC, S-UE, and S-CL activities. However, no significant interaction was detected regarding soil enzyme activities effects. These consequences suggest that the AT treatment exerted a markedly greater improvement compared to the individual application of AM or TL alone.

The two-way ANOVA uncovered that both AMF and *T. longibrachiatum* addition significantly influenced SQI, whereas no interaction effect was observed between the two factors (Figure 1e). Compared to the CK treatment, the AM and AT treatments significantly increased SQI by 52.2% and 78.9%, respectively, with AT treatment exerting the strongest positive effect on SQI. Co-inoculation with composite microbial agents demonstrated a greater enhancement of soil quality than inoculation with *T. longibrachiatum* alone.

### 3.3. Arbuscular Mycorrhizal and T. longibrachiatum on the Bacteria and Fungi Community Diversity in Albic Soil

**Figure 2:**
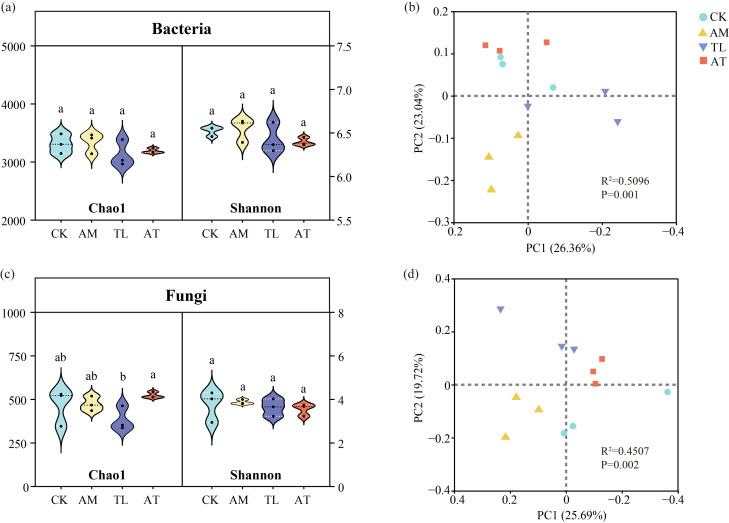
Diversity analysis of various treatments on rhizosphere microorganisms. (**a**) Bacterial α diversity; (**b**) bacterial β diversity; (**c**) fungal α diversity; (**d**) fungal β diversity. CK: Control; AM: AMF application treatment; TL: *T. longibrachiatum* application treatment; AT: AMF and *T. longibrachiatum* application treatment. Different letters showed statistically significant differences (*p* < 0.05).

No significant difference was observed in bacterial community species diversity among the treatments (Figure 2a). However, the fungal Chao1 index differed significantly between the TL and AT treatments (Figure 2c), while no statistically remarkable difference was noticed between the AM and AT treatments. Simultaneous inoculation of AMF and *T. longibrachiatum* remarkably altered fungal community species diversity compared to application of *T. longibrachiatum* as a single inoculant. PCoA and ADONIS tests revealed significant differences in microbial community constitution among treatments (Figure 2b,d), indicating that either single inoculation or co-inoculation substantially modified the microbial community structure in the rhizosphere soil.

### 3.4. Arbuscular Mycorrhizal and T. longibrachiatum on Bacteria and Fungi Community Composition

**Figure 3:**
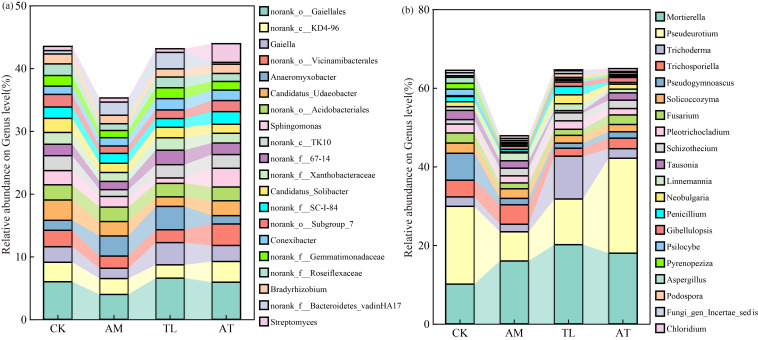
Influence of various treatments on microbial genera composition. (**a**) The abundance of bacteria; (**b**) the abundance of fungi. CK: Control; AM: AMF application treatment; TL: *T. longibrachiatum* application treatment; AT: AMF and *T. longibrachiatum* application treatment.

Norank_o_Gaiellales was the ruling bacterial genus, making up 4.83% in OTUs, and its relative amount differed significantly among the groups. Figure 3a represents the relative abundance of bacteria as a percentage of the different treatments; the relative abundance of *Anaeromyxobacter* in the TL treatment was significantly higher by 133.58% compared with the CK treatment. Similarly, the relative amount of *Sphingomonas* in the AT treatment was notably greater by 76.19% and 246.17% than in the AM and TL treatments, respectively.

*Mortierella*, *Pseudeurotium*, and *Trichoderma* were the dominant fungal genera, representing 16.20%, 15.74%, and 4.39% of the OTUs (Figure 3b), respectively. The quantity of *Mortierella* and *Trichoderma* differed significantly across treatments. Inoculation treatments significantly increased the relative abundance of *Mortierella*, with increases of 58.29%, 99.35%, and 77.86% under the AM, TL, and AT treatments, respectively. The relative abundance of *Pseudeurotium* was significantly elevated by 469.46% in the TL treatment compared to the AM treatment. *Trichoderma* reached its highest relative abundance in the TL treatment, with *T. longibrachiatum* accounting for 2.41%. Although the abundance proportions of *Pseudeurotium* improved by 22.05% in the AT treatment relative to the CK treatment, this difference was not statistically significant.

### 3.5. Arbuscular Mycorrhizal and T. longibrachiatum on Bacteria and Fungi LEfSe Differences

**Figure 4:**
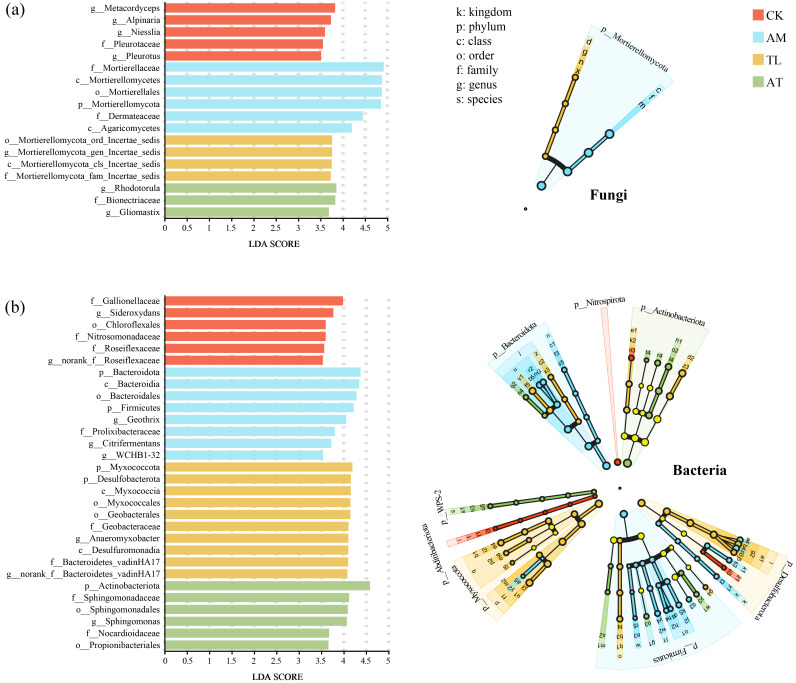
Microbial LEfSe bar and LEfSe multilevel species hierarchical tree diagrams under various treatments. (**a**) Fungal markers, (**b**) bacterial markers. CK: Control; AM: AMF application treatment; TL: *T. longibrachiatum* application treatment; AT: AMF and *T. longibrachiatum* application treatment. The figure of p, c, o, f, and g denotes phylum, class, order, family, and genus, respectively.

LEfSe differential discriminant analysis identified significantly different taxa from the phylum to genus levels, using an LDA score threshold of 3.5. As indicated by Figure 4, all 30 bacterial and 18 fungal taxa differed significantly across treatments at various taxonomic levels. Biomarker taxa within the bacterial communities varied according to the treatment group, with six biomarkers identified in the CK group, eight in AM, ten in TL, and six in AT (Figure 4a). The TL treatment exhibited the most pronounced differences, including enrichment of *Anaeromyxobacter* and norank_f_Bacteroidetes_vadinHA17. *Sphingomonas* was significantly enriched in the AT treatment. In the fungal community (Figure 4b), the AM group exhibited a maximum of differentially abundant taxa across taxonomic levels, including f_Mortierellaceae, c_Mortierellomycetes, o_Mortierellales, p_Mortierellomycota, f_Dermateaceae, and c_Agaricomycetes. In the TL treatment, significantly enriched fungal taxa were observed at the phylum, order, and class levels within *Mortierellomycota*. Fungal biomarkers in the AT treatment included *Rhodotorula*, f_Bionectriaceae, and *Gliomastix*.

### 3.6. Arbuscular Mycorrhizal and T. longibrachiatum on Microbial Function Prediction

**Figure 5:**
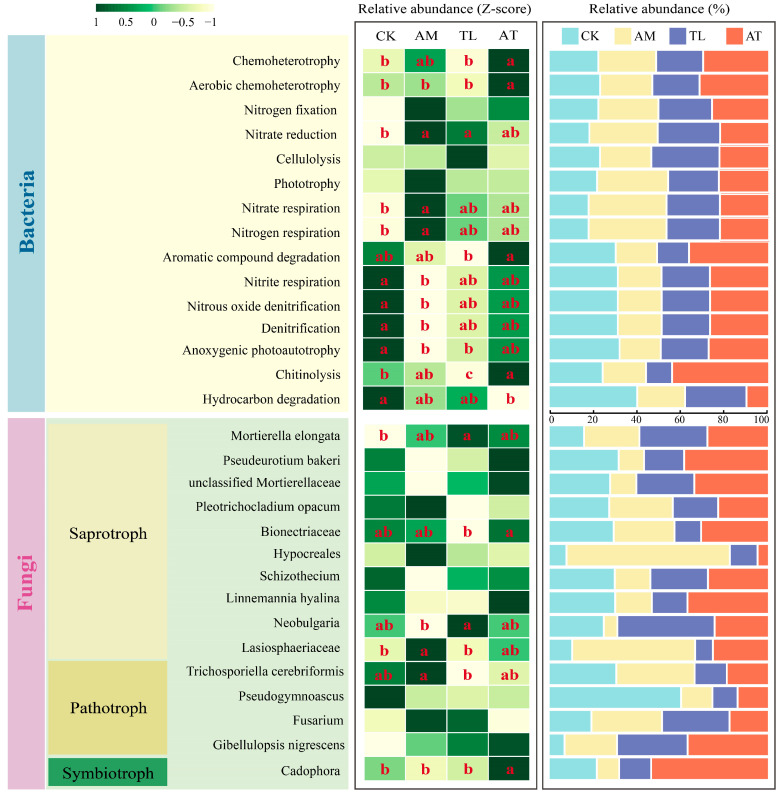
Functional prediction of bacterial and fungal communities by different treatments. Heat maps were plotted after normalization with the relative abundance Z-score. CK: Control; AM: AMF application treatment; TL: *T. longibrachiatum* application treatment; AT: AMF and *T. longibrachiatum* application treatment. Different letters in the same row show statistically significant differences.

Bacterial functional profiles differed significantly among the treatment groups (Figure 5). The AT treatment notably improved the relative amount of aerobic chemoheterotrophy, which accounted for a larger proportion of the functional bacterial community compared to treatments with single inoculants. Notably, the aerobic chemoheterotrophic activity of soil microorganisms, as well as the *Sphingomonas,* were distinctly enhanced in the AT treatment. Compared with the CK treatment, the AM significantly reduced the relative abundance of nitrite respiration, nitrous oxide denitrification, denitrification, and anoxygenic photoautotrophy, while significantly increasing the abundance of nitrate reduction, nitrate respiration, and nitrogen respiration. In contrast, the AT treatment significantly increased the relative abundance of nitrate, nitrous oxide denitrification, denitrification, and anoxygenic photoautotrophy, indicating divergent functional shifts between the treatment types.

Fungi functional guilds were analyzed using FUNGuild, which classifies fungi into three trophic modes: saprotroph, pathotroph, and symbiotroph. The saprotrophic fungus *Lasiosphaeriaceae* exhibited the highest relative abundance in the AM treatment, which was notably different from CK and TL treatments. The TL treatment markedly enhanced the proportional abundance of *Mortierella elongata* in comparison to CK. Additionally, the AT group significantly increased the quantity of the symbiotic fungus *Cadophora*.

### 3.7. Conjoint Analysis of Soil Chemical Parameters and Microbial Communities

**Figure 6:**
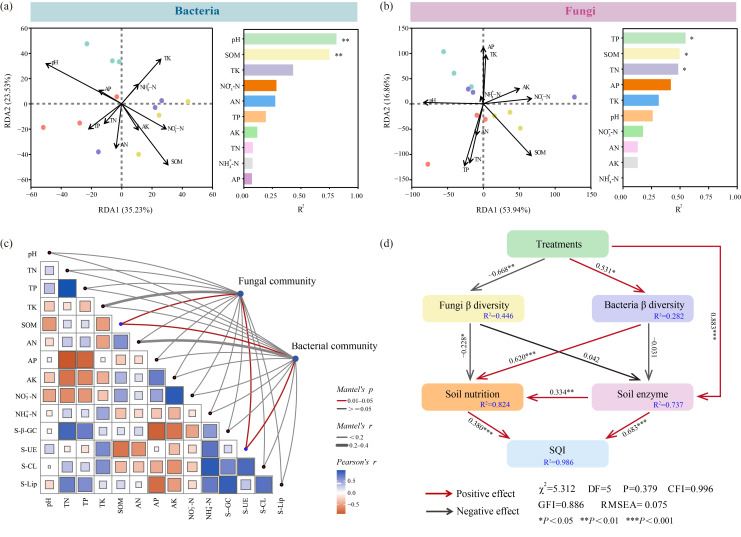
Conjoined analysis of soil parameters and microbial compositions at genus level. (**a**) RDA of bacterial communities and soil nutrients; (**b**) RDA of fungal communities and soil nutrients; (**c**) Mantel test of microbial diversity and environmental factors; (**d**) SEM analysis of microbial diversity, soil nutrients and enzymes, and SQI by various treatments, with normalization applied to each parameter and corresponding path parameters derived. Note: Mantel’s *P*: *p* value of Mantel test; Mantel’s *r*: correlation coefficient of Mantel test; Spearman’s *r*: correlation coefficient of Spearman; red indicates positive correlation and gray indicates negative correlation in SEM.

RDA showed that soil chemical properties collectively explained bacterial and fungal communities at the genus level. The genus level bacterial community demonstrated 35.23% and 23.53% to RDA1 and RDA2, with soil chemical parameters under different treatments (Figure 6a). Among these, pH (R^2^ = 0.811, *p* = 0.002) and SOM (R^2^ = 0.750, *p* = 0.003) were significantly correlated with bacterial community structure, with a negative correlation observed between pH and SOM. In Figure 6b, the RDA1 and RDA2 contributions of genus-level fungi accounted for the chemical indicators were 53.94% and 16.86% under different treatments. Significant correlations were observed between fungal community structures and TP (R^2^ = 0.548, *p* = 0.015), SOM (R^2^ = 0.498, *p* = 0.026), and TN (R^2^ = 0.483, *p* = 0.030), with all three factors showing positive correlations with one another.

There was a significant relationship between soil microbial and chemical characteristics with genus classification, with the PC1 axis of β-diversity used to explain the bacterial and fungal community composition in Figure 6c. According to Mantel test analysis, soil microbial community composition demonstrated a strong positive correlation between SOM and S-UE. Correlation analysis revealed a significant positive association between S-β-GC and both soil TN and TP. Additionally, S-CL showed a strong positive correlation with NH_4_^+^-N, while S-Lip exhibited significant proactive correlations with TN, TP, and NH_4_^+^-N. Meanwhile, TN and TP also exhibited a statistically strong positive association.

The results in Figure 6d indicate that different fungal addition treatments had an indirect but positive effect on the soil quality index (SQI), while their effects on soil enzyme activities were direct and positive. Specifically, various treatments positively influenced rhizosphere bacterial diversity and subsequently stimulated changes in soil nutrient content, ultimately leading to an improvement in SQI. In this process, the bacterial community exerted a negative effect on soil enzyme activity; however, the soil itself significantly promoted changes in nutrient availability. Additionally, both soil nutrients and soil enzyme activities had direct positive effects on SQI. These results demonstrated that different amendment treatments altered the soil microbial communities’ constituent and structure and activated soil enzyme functions, thereby promoting soil nutrient cycling and enhancing soil quality.

## 4. Discussion

### 4.1. Effects of Arbuscular Mycorrhizal and T. longibrachiatum on Chemical Properties of Albic Soils

Low-yielding characteristics such as acidic and nutrient deficient albic soils are a severe constraint to the development of agricultural productivity [34]. Previous studies have shown that inoculation with *Trichoderma* alone or AMF alone can significantly increase soil pH [35,36]. In the present study, both single and combined inoculation significantly increased the pH of albic soil, with the highest pH observed under the AT treatment. This indicates that simultaneous inoculation with AMF and *T. longibrachiatum* altered the rhizosphere microenvironment, modifying its acidity through the secretion of organic acids and promoting the proliferation of beneficial microorganisms.

The AM and AT treatments in this study significantly increased the contents of TN, TP, and TK in the soil (Table S1). Simultaneous inoculation with AMF and *T. longibrachiatum* led to a marked elevation in the concentration of S-β-GC, S-CL, and S-Lip (Figure 1). AMF are known to secrete organic acids and extracellular enzymes that promote organic matter reduction and soil nutrients accumulation [8]. *Trichoderma* can produce cellulase, β-glucosidase, and xylanase, which effectively degrade cellulose and lignin in the soil [37,38,39]. These findings found that increased extracellular enzyme activity enhanced the hydrolysis rate of recalcitrant polysaccharides. Specifically, β-glucosidase, cellulase, and lignin peroxidase facilitate the degradation of macromolecules into carbon substrates that microorganisms can readily assimilate. Consequently, this process promoted nutrient mobilization from maize stover and contributed to the accumulation of nutrients in the soil. In addition, Mantel test analysis indicated that S-β-GC was positively correlated with TN and TP (Figure 6c). The enhanced soil enzyme activity promoted the decomposition of straw organic matter and effectively accelerated soil nutrient cycling [40], which was consistent with the results from the SEM (Figure 6d). Therefore, co-inoculation with AMF and *T. longibrachiatum* outperformed single-agent treatments by synergistically enhancing extracellular enzyme secretion, improving nutrient retention, reducing nutrient loss, and significantly enhancing soil fertility in albic soils.

Notably, the TN and TP contents were considerably enhanced in the AT treatment, while the AP and AN were markedly lessened. This pattern is consistent with past findings that reported negative associations between available nutrient contents and total nutrient contents following microbial inoculation [41]. This situation put down to the truth that inoculation with both AMF and *Trichoderma* facilitated nutrient liberation from corn stover, accelerated nutrient cycling, and consequently increased the total nutrient content in the soil [7]. However, both AMF and *T. longibrachiatum* require available nutrients from the soil to support their own growth, which may lead to a reduction in the concentration of available nutrients. In addition, AMF coupled with *Trichoderma* remarkably influenced the activity of soil microorganisms, which can both mobilize otherwise inaccessible nutrients and simultaneously sequester available nutrients through microbial uptake and metabolism [42,43]. Therefore, we hypothesize that a portion of the activated available nutrients is temporarily immobilized within microbial biomass [44] and that, upon microbial death and decomposition, these fixed nutrients are released back into the soil, thereby promoting continued nutrient cycling and accumulation.

### 4.2. Effects of Arbuscular Mycorrhizal and T. longibrachiatum on Microbial Communities in Albic Soil

Both AMF and *Trichoderma* can serve as beneficial microbial agents for soil improvement by enhancing rhizosphere microbial activity and modulating the structure and function of soil microbial communities [45,46]. In this finding, the consequence of bacterial and fungal α-diversity analyses uncovered noticeable differences in fungal α-diversity between co-inoculation treatments and *T. longibrachiatum* alone. In contrast, it was discovered that there was no significant variation in co-inoculation and AMF-only treatments (Figure 2). AMF significantly enhance the development of soil mycelial networks, thereby providing additional attachment sites for *T. longibrachiatum*. Concurrently, AMF contribute to increased diversity and functional complexity within microbial communities, effects that are challenging to attain through the inoculation of *T. longibrachiatum* in isolation [47].

These dominant bacterial and fungal genera were analyzed using bar charts in Figure 3. The dominant bacterial genera included norank o Gaiellales, norank c KD4-96, and *Gaiella*, while the dominant fungal genera were *Mortierella*, *Pseudeurotium,* and *Trichoderma*. Notably, the AT signally enhanced the proportion of *Sphingomonas* in comparison with the AM and TL treatments (Figure 3a), and *Sphingomonas* was also confirmed as a vastly enriched taxon in the AT treatment (Figure 4a). Previous studies have demonstrated that AMF and *Sphingomonas* can synergistically improve petroleum degradation efficiency, thereby facilitating phytoremediation of contaminated soils [48]. In addition, *Sphingomonas* is capable of producing siderophores, which play a crucial role in metal chelation [49]. It also contributes to alleviating herbicide toxicity and residues in soil, participates in plant–microbe co-metabolic degradation, and promotes in situ remediation of soil pollutants [50]. *Sphingomonas* is a keystone species in the AT treatment. *Sphingomonas* acts as a “super-decomposer” within interactions involving AMF and *T. longibrachiatum*. It facilitated the mobilization of insoluble nutrients in the soil, thereby established a reciprocal mechanism characterized by “bacterial activation and fungal transport” among *Sphingomonas*, AMF, and *T. longibrachiatum*.

*Trichoderma*, as a dominant genus of fungi, exhibited a significant increase in relative abundance in the TL treatment group, highlighting the ability of *T. longibrachiatum* to form associations with AMF and successfully colonize soybean roots. Existing studies are consistent with our findings, showing that *Pseudeurotium* and *Trichoderma* are key fungal taxa involved in humification, capable of secreting various extracellular enzymes and efficiently degrading cellulose and lignocellulose [51]. This research discovered that the relative amount of *Pseudeurotium* decreased under the AM and TL treatments but was substantially increased and greatest in the AT treatment (Figure 3b). These findings suggest that the combination of AMF and *T. longibrachiatum* can modulate microbial ecological competition, enhance extracellular enzyme secretion, and reshape the rhizosphere microenvironment.

### 4.3. Effects of Arbuscular Mycorrhizal and T. longibrachiatum on Soil Quality of Albic Soils

Soil microbial activity and community changes can enhance interactions among microorganisms, thereby affecting their physiological and biochemical processes and effectively improving soil quality. Previous studies have found that inoculation with *Trichoderma* and AMF introduces beneficial microorganisms into the soil, stimulates functional microbial metabolic activity, and increases rhizosphere nutrient content [52,53]. In this study, AMF joined with *T. longibrachiatum* remarkably enhanced the albic soil quality and notably increased the constituents of TN, TP, and TK in the AT treatment (Table S1). Inoculation with AMF or *Trichoderma* alone can activate beneficial microorganisms, increase soil pH, and improve the acidic soil environment [54,55]. This study found that the AT treatment remarkably improved soil pH, and the bacterial community significantly correlated with pH (Figure 6a). This study demonstrated that the alleviation of soil acidification through the combined action of AMF and *T. longibrachiatum* primarily stemmed from the enhancement of aerobic chemoheterotrophy activity dominated by *Sphingomonas*. Additionally, inoculation with AMF and *Trichoderma* can expand the root zone, increase contact between soil microorganisms and the root system, enhance the functional expression of microorganisms, and further improve rhizosphere soil quality [55,56].

The amount of chemoenergetically heterotrophic bacteria increased in the AT treatment (Figure 5). Bacterial communities with nitrification functions have been reported to elevate soil pH, while chemoenergetic heterotrophs enhance soil nutrient availability and modulate biogeochemical processes [57]. *Sphingomonas*, a differential genus enriched in the AT treatment, is able to produce plant growth hormones that improve plant stress resistance and promote plant growth [58,59]. Microbial inoculants, particularly those with synergistic plant growth-promoting capabilities, make a critical difference in adding soil nutrient accumulation and elevating overall soil quality [60].

While the functional prediction outcomes offer important insights into the potential roles of microbial communities, these predictions are derived from phylogenetic marker genes and should be interpreted as hypotheses rather than definitive conclusions. The precise metabolic functions of *Sphingomonas* identified in this study require validation through more comprehensive metagenomic sequencing. Furthermore, microbial sequencing methodologies may fail to detect rare yet functionally significant microorganisms within the community. Consequently, the functional predictions presented in this study are likely biased toward taxa exhibiting relatively high abundance in the AT treatments.

In the present study, the combined AMF and *T. longibrachiatum* treatment was more effective in improving both soil enzyme activity and the SQI compared to individual inoculations. *Trichoderma* secretes extracellular enzymes that degrade soil macromolecules and produce antibiotics to suppress pathogens. The hyphal network of AMF provides extensive sites for *Trichoderma* colonization and movement, amplifying and extending its biological effects and activity. *Trichoderma* acts as a “pioneer modifier of the soil chemical environment”, while AMF serves as an “amplifier of nutrient acquisition”. The co-inoculation significantly influenced soil nutrient dynamics by restructuring the components and functional aspects of bacterial communities and exhibited the strongest effect on albic soil quality enhancement. SEM further indicated that soil enzyme activity directly improved SQI and indirectly enhanced it by accelerating nutrient cycling. Therefore, dual inoculation with AMF and *T. longibrachiatum* not only modified soil microbial ecological functions but also increased enzyme activity, facilitated nutrient accumulation, and substantially elevated soil quality.

## 5. Conclusions

Albic soil is a typical form of degraded soil characterized by acidification, severe physical structural barriers, poor nutrient retention, and low microbial activity. This study demonstrated that the combination of AMF and *T. longibrachiatum* significantly increased the activities of S-β-GC, S-UE, and S-Lip, as well as soil pH, thereby enhancing extracellular enzyme-mediated straw decomposition and increasing the total nutrient content in the soil. The interactions between AMF and *T. longibrachiatum* also markedly increased the relative abundance of the differential genus *Sphingomonas* and the population of aerobic chemoheterotrophic bacteria, consequently altering the functional composition of the microbial community. Moreover, co-inoculation with AMF and *T. longibrachiatum* significantly improved the SQI of albic soil, demonstrating that this strategy is an effective biotechnological approach to improving soil quality. Therefore, the combined application of AMF and *T. longibrachiatum* holds great promise for the remediation of albic soils under field conditions; however, its sustainability and practical applicability require validation through long-term field studies.

## Data Availability

The original data presented in the study are openly available in the NCBI BioProject. The accession number is PRJNA1334601, and the direct link is https://www.ncbi.nlm.nih.gov/bioproject/PRJNA1334601/ (accessed on 17 January 2025).

## Supplementary Materials

The following supporting information can be downloaded at: https://www.mdpi.com/article/10.3390/jof11100747/s1, Figure S1: Microbial LEfSe multilevel species hierarchical tree diagrams under various treatments; Figure S2: Map of the experimental location; Table S1: Two-way ANOVA analysis of different treatments on soil chemical properties.
