# Supplementary material for: Arbuscular Mycorrhizal and Trichoderma longibrachiatum Enhance Soil Quality and Improve Microbial Community Structure in Albic Soil Under Straw Return
Source: J Fungi (Basel). 2025 Oct 18;11(10):747. doi: 10.3390/jof11100747 (PMC12565002; doi:10.3390/jof11100747)
Supplement: Supplementary file 1 [file jof-11-00747-s001.zip › jof-3913389-supplementary.pdf]

(a)

Fungi

a : p__Mortierellomycota
c : c__Mortierellomycetes
d : c__Mortierellomycota_cls_Incertae_sedis
f : o__Mortierellales
g : o__Mortierellomycota_ord_Incertae_sedis
m : f__Mortierellaceae
n : f__Mortierellomycota_fam_Incertae_sedis
x : g__Mortierellomycota_gen_Incertae_sedis

CK
AM
TL
AT

(b)

Bacteria

q2 : f__Prevotellaceae
w2 : f__Thermincolaceae
y2 : f__Vulgatibacteraceae
e3 : f__norank_o__Clostridia_UCG-014
i3 : g__Abditibacterium
m3 : g__BSV13
o3 : g__Caproiciproducens
q3 : g__Clostridium_sensu_stricto_8
s3 : g__Desulfobulbus
u3 : g__Desulfurivibrio
y3 : g__Fonticella
a5 : g__Vulgatibacter
k5 : g__norank_f__Hungateiclostridiaceae
s5 : g__norank_o__Kapabacteriales
u5 : g__norank_p__WPS-2
l5 : g__norank_f__Nannocystaceae
r5 : g__norank_o__Clostridia_UCG-014
o2 : f__Nocardioidaceae
b : p__Actinobacteriota
d : p__Desulfobacterota
f : p__Myxococcota
h : p__WPS-2
j : c__Bacteroidia
l : c__Desulfuromonadia
n : c__Kapabacteria
p : c__Myxococcia
t : o__Abditibacteriales
x : o__Desulfovibrionales
z : o__Flavobacteriales
b1 : o__Haliangiales
f1 : o__Myxococcales
h1 : o__Propionibacteriales
l1 : o__Thermincolales

t1 : f__Abditibacteriaceae
v1 : f__Bacteroidetes_vadinHA17
x1 : f__Desulfobulbaceae
z1 : f__Desulfurivibrionaceae
d2 : f__Gaiellaceae
h2 : f__Gracilibacteraceae
j2 : f__Hungateiclostridiaceae
p2 : f__Phaselicystidaceae
r2 : f__Prolixibacteraceae
x2 : f__Thermoactinomycetaceae
b3 : f__norank_c__Limnochordia
f3 : f__norank_o__Kapabacteriales
h3 : f__norank_p__WPS-2
j3 : g__Anaeromyxobacter
m4 : g__Phaselicystis
u4 : g__Roseisolibacter
y4 : g__Thermincola
g4 : g__Lachnoclostridium
n3 : g__CL500-29_marine_group
p3 : g__Citrifermentans
t3 : g__Desulfovibrio
x3 : g__Flavobacterium
z3 : g__Gaiella
b4 : g__Geotalea
d4 : g__Gracilibacter
f4 : g__Hydrogenispora
h4 : g__Lutispora
l4 : g__Nocardioides
r4 : g__Pseudonocardia
t4 : g__Rhodococcus
b5 : g__WCHB1-32
f5 : g__norank_f__Bacteroidetes_vadinHA17
j5 : g__norank_f__Geobacteraceae

a : p__Abditibacteriota
c : p__Bacteroidota
e : p__Firmicutes
g : p__Nitrospirota
i : c__Abditibacteria
k : c__Desulfovibrionia
o : c__Limnochordia
q : c__Polyangia
s : c__norank_p__WPS-2
u : o__Bacteroidales
w : o__Clostridia_UCG-014
a1 : o__Geobacterales
c1 : o__Kapabacteriales
e1 : o__Microtrichales
g1 : o__Oscillospirales
m1 : o__Thermoactinomycetales
w1 : f__Caloramatoraceae
y1 : f__Desulfovibrionaceae
c2 : f__Flavobacteriaceae
s1 : o__norank_p__WPS-2
u1 : f__Anaeromyxobacteraceae
m2 : f__Myxococcaceae
a4 : g__Geobacter
e4 : g__Haliangium
o1 : o__norank_c__Clostridia
q1 : o__norank_c__Limnochordia
g2 : f__Geobacteraceae
i2 : f__Haliangiaceae
k2 : f__Ilumatobacteraceae

Figure S1. Microbial LEfSe multilevel species hierarchical tree diagrams under various treatments. (a) fungal markers (b) bacterial markers. CK: Control; AM: AMF application treatment; TL: *T. longibrachiatum* application treatment; AT: AMF and *T. longibrachiatum* application treatment. The figure of p, c, o, f and g denotes phylum, class, order, family and genus respectively.

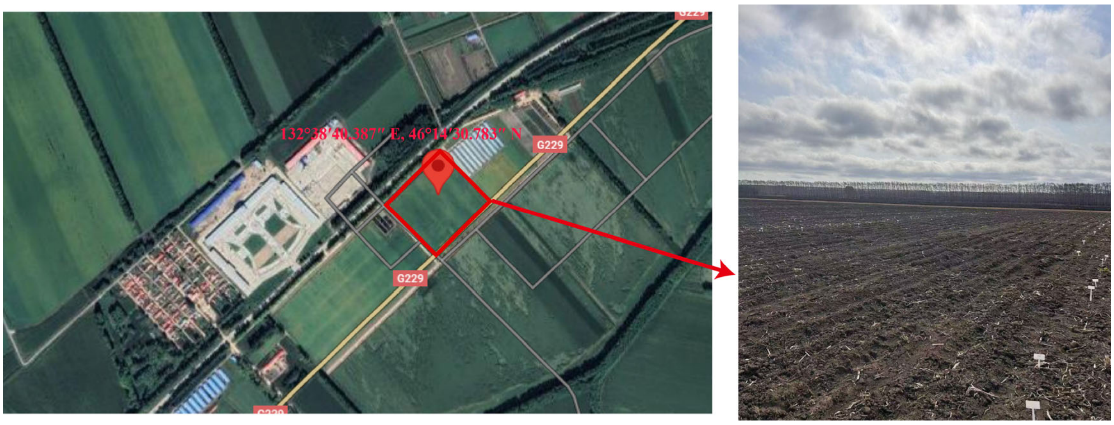

Figure S2. Map of the experimental location. The latitude and longitude of the map are the exact locations of the experimental.

Table S1. Two-way ANOVA analysis of different treatments on soil chemical properties.

Treatments	CK	AM	TL	AT	*F*(AM)	*F*(TL)	*F*(AM×TL)
pH	6.06±0.02d	6.24±0.03b	6.11±0.03c	6.29±0.03a	159.01**	10.12**	0.01ns
SOM/($g \cdot kg^{-1}$)	35.70±0.15a	33.83±0.25b	35.33±0.32a	35.67±0.40a	18.44**	20.15**	41.49**
TP/($g \cdot kg^{-1}$)	0.64±0.01d	0.66±0.01c	0.74±0.02a	0.71±0.01b	0.23ns	163.17**	21.94**
TK/($g \cdot kg^{-1}$)	15.17±0.06c	15.77±0.06a	14.90±0.00d	15.60±0.00b	760.50**	814.5**	4.50ns
TN/($g \cdot kg^{-1}$)	1.46±0.11d	1.57±0.11c	1.77±0.01a	1.74±0.01b	41.14**	1522.57**	126.00**
AP/($mg \cdot kg^{-1}$)	21.97±0.40a	21.33±0.35a	20.37±0.35b	19.83±0.35b	7.66*	54.06**	0.06ns
AK/($mg \cdot kg^{-1}$)	161.33±0.58a	108.67±0.58c	114.67±0.58b	107.33±0.58d	8100.00**	5184.00**	4624.00**
AN/($mg \cdot kg^{-1}$)	130.67±2.31a	127.00±4.58a	131.33±0.58a	130.00±0.00a	1.80ns	2.45ns	1.25ns
NO_3^--N/($mg \cdot kg^{-1}$)	3.89±0.48a	2.01±0.20b	2.22±0.22b	2.46±0.25b	21.35**	11.83**	35.64**
NH_4^+-N/($mg \cdot kg^{-1}$)	0.93±0.09a	1.03±0.10a	0.95±0.09a	1.06±0.11a	3.45ns	0.17ns	0.01ns

CK: Control; AM: AMF application treatment; TL: *T. longibrachiatum* application treatment; AT: AMF and *T. longibrachiatum* application treatment. Values in the table are presented as means ± standard error. Different letters in the same row showed a statistically significant differences. *$P < 0.05$; ** $P < 0.01$; ns: No statistically significant difference.
